# Managing understandings of palliative care as more than care immediately before death: Evidence from observational analysis of consultations

**DOI:** 10.1111/hex.13903

**Published:** 2023-11-05

**Authors:** Holly Sansone, Stuart Ekberg, Sarah Lord, James Stevenson, Katherine Martinez, Patsy Yates

**Affiliations:** ^1^ School of Psychology and Counselling Queensland University of Technology Kelvin Grove Queensland Australia; ^2^ The Prince Charles Hospital Chermside Queensland Australia; ^3^ Faculty of Health Queensland University of Technology Kelvin Grove Queensland Australia

**Keywords:** clinical interactions, conversation analysis, death, palliative care, professional–patient relations

## Abstract

**Background:**

Evidence suggests that public, and some professional, understandings of palliative care are limited to care provided immediately before death, which contrasts palliative care's scope as care provided across a range of illness stages.

**Objective:**

To examine how clinicians manage patients' understandings of palliative care during initial consultations.

**Design:**

Initial palliative care consultations were video‐recorded and analysed using conversation analytic methods.

**Setting/Participants:**

Consultations were recorded in a specialist palliative care outpatient unit within an Australian public hospital. Participants included 20 newly referred patients and their families, and three palliative care clinicians.

**Results:**

During initial consultations, it was observed that specialist palliative care clinicians frequently managed the possibility that patients may understand palliative care as limited to care provided immediately before death. Clinicians used recurrent practices that seemed designed to pre‐empt and contradict patients' possible narrow understandings. When discussing the palliative care inpatient unit, clinicians recurrently explained inpatient care could include active treatment and referred to the possibility of being discharged. These practices contradict possible understandings that future admission to the inpatient unit would be solely for care immediately before death.

**Discussion:**

The findings demonstrate that palliative care clinicians are aware of possible narrow understandings of their discipline among members of the public. The practices identified show how clinicians pre‐emptively manage these understandings to patients newly referred to palliative care.

**Conclusions:**

These findings highlight scope for greater partnership with teams referring patients to palliative care, to assist patients in understanding the range of reasons for their referral.

**Patient or Public Contribution:**

The observational method of conversation analysis provides direct insight into matters that are relevant for patients, as raised in their consultations with clinicians. This direct evidence enables analysis of their lived experience, as it occurs, and grounds analysis in observable details of participants' conduct, rather than interpretations of subjective experiences. The patients' contributions, therefore, were to allow observation into their initial palliative care consultations.

## 1 INTRODUCTION

A holistic focus is well established within the palliative care profession and among leading advocates for palliative care development,^1^ such as the World Health Organization.^2^ Palliative care includes pharmacological, psychological and procedural treatments to reduce symptoms across a range of illness stages,^3^ including alongside other treatments aimed at disease stabilisation or cure, so‐called ‘active treatments’. Beyond the palliative care profession and advocacy groups, however, understandings of palliative care overwhelmingly remain synonymous with death‐focused concepts, including cessation of treatment,^4^ care provided only in the last months of life,^5^ hopelessness and dependency^6^ and as a euphemism for death.^7^ Although these understandings may be consistent with the deteriorating and terminal phases of palliative care for chronic illnesses and cancer, they do not easily equate with the very long periods people can receive palliative care while their condition is stable. Evidence suggests that some referring clinicians may be insufficiently equipped to manage patients' understandings of palliative care as more than care provided immediately before death,^8^, ^9^, ^10^ while some also hold these views.^11^, ^12^ As well, some government policies may reinforce these limited understandings of palliative care by placing eligibility for palliative care at the point when a patient's primary goals become comfort,^13^ rather than coinciding with active or other life‐prolonging treatments.^2^, ^14^, ^15^

In Australia, as many as 90% of people surveyed report having heard of palliative care.^16^ Despite this general awareness, misunderstandings about palliative care persist. For instance, only 40% of Australians who report knowing about palliative care identify it as appropriate for those newly diagnosed with a life‐threatening or life‐limiting illness.^16^ Similar trends are shown in other countries. For instance, a survey in Northern Ireland found 83% of people knew of palliative care but the majority (75%) associated it with terminal care.^17^ These existing understandings of public perceptions about palliative care are based on self‐report data and generated through methods such as surveys and focus groups.^18^ The findings of this research suggest that recently referred community‐dwelling patients may come to palliative care with misunderstandings about the care they can receive. The current study is designed to understand how possible understandings of palliative care are displayed and managed by both patients and clinicians during initial palliative care consultations.

Discussions about matters relating to life‐threatening and life‐limiting illness are known to be difficult and delicate.^19^, ^20^ Lack of training in how to conduct these difficult discussions is reported by treating clinicians as a barrier for early referral to palliative care,^21^, ^22^, ^23^ despite evidence that early referral enhances quality of life.^24^, ^25^ Research suggests that palliative care clinicians are more likely to discuss death and dying with families than other healthcare professionals.^26^ It is beneficial, therefore, to explore how specialist palliative care clinicians conduct these delicate discussions with newly referred patients, and how they might manage patients' understandings of palliative care.

A small but growing body of research has explored interactions between palliative care clinicians, patients and their caregivers, such as those relating to prognosis^27^, ^28^, deterioration and dying^29^, ^30^, ^31^, ^32^ and assurance and reassurance.^33^, ^34^ None of this research, however, has focused on communication in initial outpatient palliative care consultations. This article extends existing interactional research by exploring how specialist palliative care clinicians manage newly referred patients' understandings of palliative care in their initial consultations.

## 2 METHOD

This qualitative study used observational methods, video‐recording initial palliative care consultations involving clinicians, patients and sometimes family members, to generate direct evidence of communication practices used during patients' initial interactions with palliative care. These methods allow analysis to be grounded in real‐life conversations, rather than relying on hypotheses or theoretical constructs.^35^

Data were collected from a specialist palliative care outpatient clinic of an Australian public hospital. Consultations were recorded only if every person involved independently provided informed, written consent to participate.

### 2.1 Setting

Recorded consultations were conducted from March to December 2021. Patient participants were newly referred from either their general practitioner (GP) or from a clinical area within the hospital, indicated by primary diagnosis (see Table 1). Average Charlson comorbidity index within the patient cohort was 6,^36^ signifying very high predicted mortality at 1 year. Two specialist palliative healthcare professionals, one palliative specialist trainee (registrar) and one administrative officer, were present in one or more of the recorded consultations. Most often, only one palliative healthcare professional was present with the patient and family during the consultation.

**Table 1:** Demographic characteristics of newly referred palliative care patients.

Characteristics	
Participants	*n* = 20
Gender	
Male	13 (65%)
Female	7 (35%)
Age	
Mean	69 years
Range	45–91 years
Primary diagnosis	
Cancer	16 (80%)
Lung	7
Pancreatic	2
GI tract	2
Breast	1
Colorectal	1
Other (GBM, SCC, prostate)	3
Metastatic disease present	15 (94%)
Lung disease	2 (10%)
Other (MND, ESRF)	2 (10%)
Charlson comorbidity index	
Mean	6.45
Median	6
Range	0–10
Mobility	
Independent	15 (75%)
Four‐wheeled walker	3 (15%)
Walking stick	1 (5%)
Other	1 (5%)
Nationality	
Australian	11
United Kingdom	4
Ireland	2
Other (New Zealand, South Africa, India)	3
English as primary language	20 (100%)
Initial consultation attended by patient and a support person(s)	16 (80%)

Abbreviations: ESRF, end stage renal failure; GBM, glioblastoma multiforme; MND, motor neuron disease; SCC, squamous cell carcinoma.

### 2.2 Data collection

Three specialist palliative care clinicians and two specialist trainees were approached to participate, and of these, two clinicians and one trainee gave informed, written consent. Families involved in the recordings were purposefully selected and informed about the project by a hospital staff member. Twenty patients and their families provided informed, written consent for their consultation to be recorded, creating 20 recordings of initial consultations. Participants could indicate whether they would like their image blurred, voice changed and/or their name removed from the recorded data, or indicate that they do not require any of these.

Before the commencement of the initial consultation, a member of the hospital staff would set up two video cameras in the consultation room, receive participants' consent and begin recording. No researchers were present during these consultations. All recordings were uploaded immediately to a secure server and then held securely at the research institution. A confidentiality agreement was required from those transcribing the audio files of the recordings. Names within the reproduced transcripts have been pseudonymised.

### 2.3 Data analysis

The data were analysed using the inductive, observational approach of conversation analysis.^35^ This methodology was selected as it examines real‐world interactions to understand how specific interactional practices are used to accomplish recognisable actions,^37^ rather than relying on more indirect evidence such as surveys or interviews, and is widely used for healthcare communication research.^38^, ^39^

#### 2.3.1 Rigour

Despite all participants knowing and consenting to being recorded, evidence suggests that recorded data is a reasonably true representation of what transpires during consultations without the recording instruments in place. For instance, clinician behaviour during consultations has been demonstrated to show no significant differences whether the clinician was aware or unaware of being recorded.^40^, ^41^ Another study demonstrated that approximately 70% of patients reported they ‘forgot’ the camera was present during recorded consultations.^42^ This evidence supports the likelihood that the data collected for this study is reasonably representative of consultations held within the palliative care outpatient clinic.

Conversation analysis grounds analysis in observable aspects of social conduct.^37^ In doing this, it ensures that the analysis is based on participants' experiences rather than on a priori theorising that relies upon researchers' presumptions and intuitions.^35^ Recorded instances are meticulously transcribed using conventions that capture details within vocal conduct, such as sound stretches, silences and other relevant features,^43^, ^44^ to facilitate the fine‐grained, turn‐by‐turn, examination of each instance. A glossary of transcription conventions used within this article is available (Supporting Information: Appendix A).

This data‐driven analysis consists of examining transcripts alongside recorded data to build an exhaustive collection of a particular communication practice of interest. Analytical interpretation is subjected to a check of validity by next‐turn proof procedure.^35^, ^45^ This is the act of comparing any claim made by the analyst to how interactants themselves indicate their understanding of a prior turn within the conversation.^35^, ^45^ Rigour was further maintained by working collaboratively with conversation analysts, specialist palliative care clinicians and palliative care researchers for reliability and reflexivity checks.

## 3 ANALYSIS

During consultations with newly referred patients, specialist palliative care clinicians often have many topics to discuss, such as those relating to symptom management, patients' ongoing care and their available supports (e.g., family who live locally). In 60% of initial consultations recorded, the clinician also introduced the three overarching ways their service could provide palliative care to the patient: (1) the outpatient clinic, which was where these consultations took place; (2) community‐based palliative care, reserved for those who could no longer leave the house; and (3) inpatient palliative care. During these consultations, the clinicians also frequently managed the possibility that patients understand palliative care as being synonymous with care provided immediately before death. An analysis of this follows, using exemplars from the collected instances, and is organised into two parts. First, consideration is given to understanding patients' displays about palliative care being synonymous with end‐of‐life care. Such understandings were most often displayed in relation to the care delivered in the specialist inpatient palliative care unit. Second, consideration is given to ways clinicians pre‐empt the possibility of these narrow understandings of palliative care.

Understandings of palliative care as relating to death and death‐related concepts are observable in patients' questions about the inpatient palliative care unit. The following two fragments provide examples of how patients demonstrate their narrow understanding of palliative care when inpatient palliative care is introduced. In Fragment 1, a patient displays an understanding of palliative care being narrowly focused on care provided immediately before death, while Fragment 2 contains a more indirect display of the patient's understanding.

Fragment 1 begins with the clinician explaining that the frequency of contact between the patient and palliative care team will be responsive to the patient's needs:

**Fragment 1 [P14/E1/14:35–15:03]**

01 CLI And sometimes you don't need to see each other

02 for months that's okay?

03 PAT Right.

04 CLI Sometimes I need to see you every 2 weeks.=especially

05 if you have symptoms that I'm trying to get on top of.

06 PAT I see.

07 CLI Okay?

08 CLI So:.hhh we do have this cli:nic¿.hh we have a sixteen‐

09 bed unit, (.) at‐ ah on the other side of this wa:ll really.

10 PAT **So that's for the (.) very late sta:ge, >type of thing.<**

11 CLI ↑Yeah↓ I also admit people with um, (.) say pain crisis.

12 PAT P‐ Pardon¿

13 CLI Pain crisi::s?

14 PAT Okay.

The fragment begins with the clinician explaining possibilities for their frequency of contact. The first possibility is they would not need to see each other for months (lines 1 and 2), presumably because the patient is feeling well. The second possibility is they could need to see each other every 2 weeks (line 4). The clinician explains this greater frequency of contact would be to actively provide treatment to manage the patient's symptoms. The patient acknowledges this information (line 6). After the clinician mentions the inpatient unit (lines 8 and 9), however, the patient offers a candidate understanding^46^ of the inpatient palliative care unit as being for the ‘very late stage’ (line 10). This candidate's understanding is presented as a declarative question,^47^ which allows the clinician to either confirm or disconfirm the patient's understanding that the inpatient unit is a place solely for care provided immediately before death. The patient refers to this indirectly, ‘very late stage type of thing’ (line 10). Indirect references to death can be common in palliative care consultations.^29^ This fragment displays how this patient's concept of a hospital‐based palliative care unit is synonymous with approaching death.

The clinician confirms the patient's understanding but then explains that another use for the inpatient unit is to manage problems such as pain (line 11). Through the use of ‘also’ (line 11), the clinician emphasises that palliative care includes active treatment in addition to care provided immediately before death (line 11). The patient accepts this explanation (line 14). This fragment illustrates the type of narrow understandings of palliative care patients that can display during initial palliative care consultations, how it can shape the conversation and how clinicians must manage patients' understandings.

Fragment 2 is from another initial consultation. Here, the clinician and patient are discussing the patient's currently unmanaged pain. Before the fragment begins, the clinician explains that the patient is likely experiencing two types of pain, which may require progressively introducing multiple medications:

**Fragment 2 [P5/E1/11:35–12:10]**

01 CLI So::, i:f after that, (0.8) pai:n, is still really ba:d¿

02 then we probably need to talk about getting, you i:nto ou:r,

03 (0.6) ward.=i[nto our] unit,.hhh for <pain management.>=

04 PAT. [ mmm,]

05 PAT =Yeah.=

06 CLI =So that means,.hhh if you're ther:e, ((points in direction

07 of the inpatient unit)) and a doctor is there every

08 da:y, we can make (.) changes quicker.

09 PAT Yep. [Okay,]

10 CLI [.hh] We can get you to a better: (0.4) mor‐

11 more comfortable spa::ce,

12 (.)

13 CLI quicker¿

14 PAT Mm hm,

15 CLI And if we need to do other thi:ngs, (.) you

16 kn[ow, we]can do it in [the hospital.]

17 PAT [a::nd,] **[And thE:n I c'n] go**

18 **home if I get‐ (0.4)**

19 CLI De:finitely.=

20 PAT =So it's not [(you know)]

21 CLI [M'kay¿,] so it's <↑N:Ot a O:ne

22 way (.) [doo:r?>]

23 PAT [ Yep.]

24 PAT >That's good.< Okay.

In lines 1–3, the clinician suggests the possible use of the palliative care unit for active treatment, to manage the patient's pain. The clinician emphasises the purpose of admission by slowing down the rate of their speech when saying ‘pain management’ (line 3). The clinician's suggestion of actively managing the patient's condition alludes to the possibility of their improvement. Following the clinician's elaboration of possibly needing to do ‘other things’ while in hospital (line 15), which suggests extending the hospital admission, the patient seeks clarification. Specifically, they ask if they would be able to go home under certain circumstances, though those circumstances remain unspecified (lines 17 and 18). Following the clinician's definitive confirmation of this (line 19), the patient begins to ask what the admission is not about (line 20). Even before the patient's question is complete, the clinician responds to dismiss the possibility that patients are only admitted but never discharged from the inpatient unit (lines 21 and 22). The patient displays being assured by the clinician's remarks by responding with an assessment (‘That's good’, line 24).

These two fragments illustrate how patients attending initial palliative care consultations can invoke understandings of palliative care as care provided immediately preceding death, even when there is discussion of other treatments offered by the palliative care team (e.g., active treatment of pain). Patients' responses to clinicians' talk about the inpatient palliative care unit expose these understandings, which shape the ensuing interaction. For example, in Fragment 2 the patient's urgency to clarify their understanding is displayed by their overlapping the clinician's talk (line 17). The data examined in Fragments 1 and 2 demonstrate how patients can display understandings, specifically that admission to inpatient palliative care is synonymous with care provided immediately before death. These fragments highlight the importance of managing patients' expectations about palliative care around the time of referral. Instances such as this were relatively rare in the data collected for this study because often clinicians used practices that seemed designed to pre‐emptively contradict potential understandings that the inpatient unit is solely for care provided immediately before death. Clinicians recurrently used two practices designed in a particular way to achieve this. Analysis of the next two fragments will explore these practices.

The first practice involves clinicians using extended turns‐at‐talk^45^ to introduce and explain the inpatient palliative care unit as having multiple functions. The unit is described as providing care additional to care immediately before death, including active treatment of symptoms. The second practice involves clinicians referring to the possibility of being discharged from the inpatient unit. Considering the types of understandings that were displayed by patients in Fragments 1 and 2, these practices seem designed to manage new patients' understandings of palliative care and expectations of the palliative inpatient unit. Additionally, these two practices were recurrently designed using generalisations or nonperson‐specific terms. When speaking about care offered by the palliative care service, clinicians referred to ‘people’ generally rather than the patient specifically. As will be shown, generalisations provide means to pre‐empt and contradict possible understandings about palliative care, but without claiming that the patient necessarily subscribes to them.

The next fragment begins following the clinician's explanation of community‐based palliative care, and why this was currently unnecessary for the patient. The clinician then explains the inpatient unit. The focal practices are indicated using numbered arrows.

**Fragment 3 [P2/E1/21:59–22:34]**

01 CLI In terms of the palliative care wa:rd, ah:m, (0.4)

02 so:, (0.2) of course the ward is there for e:nd of life,

03 (0.4)

04 CLI **1**→ but >**a lot of people don't know it's also there for**

05 **1→ symptom management< as well.=so sometimes people come in**

06 **1→ because they've got distressing symptoms**,

07 (0.4)

08 CLI and try‐ we try to get on top of tha:t,

09 (0.4)

10 CLI **2→** an once it's c'ntrolled **we get them back ho::me**

11 or, (0.4) wherever they want to go from there.=So:,

12 **1→ we also try to (0.2) admit people to help out with any symptoms¿**

13 (0.6)

14 CLI if required as well. so jus’ (.) so you know that kind

15 of (0.2) option is the:re if you have any problems,

16 PAT Yeah.

17 CLI in the futu[re.]

18 PAT [Ye]h.

19 (1.0)

20 CLI Do you have any questions at all?

21 (0.6)

22 PAT N:ah, no.

The clinician's extended turn‐at‐talk begins by acknowledging that palliative care involves care at the end of life. This statement addresses the possibility that the patient may understand the inpatient unit being provided to people immediately before death (line 2). The clinician's expansion of their turn, however, provides an explanation that contradicts a potential understanding of the inpatient unit as restricted to end‐of‐life care. The clinician refers to people generally (lines 4 and 5), while explaining reasons for being admitted to the inpatient unit beyond care delivered immediately before death. In this case, the clinician reports that ‘people’ may experience difficult symptoms that can be managed in the unit (arrow 1, lines 4–6), which foregrounds that inpatient palliative care can include active treatment. The clinician next explains that patients are discharged home once symptoms are stable (arrow 2, line 10). This information contradicts a possible understanding that patients admitted to the inpatient palliative care unit always remain there until their death.

Across the fragment, the clinician speaks in general terms about ‘people’ (lines 4, 6 and 12) before directing talk to the patient individually (lines 14 and 15). This characterisation of people generally softens the direct relevance of the matter to the recipient.^48^, ^49^ It provides a way for the clinician to contradict a possible understanding of palliative care being narrowly focused on care provided immediately before death, but without claiming that this patient necessarily holds such a view.

The next fragment demonstrates the same practices seen in Fragment 3 and how they are recurrently used to contradict possible understandings of inpatient palliative care as synonymous with care provided immediately before death. During this fragment, the patient does not respond verbally to the clinician. Attempts to transcribe whether and how the patient may have responded nonverbally to the clinician, such as through nodding, was not possible due to the camera angle and his wearing a hat. Before the consultation began, this patient had accidentally walked into the inpatient palliative care unit instead of the outpatient clinic. This accidental visit to the inpatient unit is referred to in the first two lines of the fragment below (lines 1 and 2). During the consultation, immediately before the fragment began, the clinician is explaining different aspects of the service. The following explanation of the inpatient unit comes after an explanation of community‐based palliative care:

Fragment 4 [P7/E1/26:07–27:30]

01 CLI .Hhhh Now. the other thing that we do is (.) eh you've

02 been ther:e, the other side of the:, this wall¿.hhh

03 that's our palliative care unit.

04 (.)

05 CLI So we've got a sixteen bed uni:t? (0.8) that

06 **1→ we take care of pe:ople at various sta:ges of their journey.=**

07 **1→ of their palliative care journeys.**=Some of them will be end

08 of li:fe? Half of them would be end of li:fe?.hhhh a third

09 **1→ would be in there because they've got so:: much symptoms,**

10 **1 → .hhh we need to change their medications every day.**

11 (0.4)

12 CLI And it's not just gonna happen quickly if we are (0.2)

13 managing them a‐ from home.=So we have to bring them in

14 there, change things, change them again tomorrow, and then

15 **2→ get them ho:me onc:e, (.) so we (0.2) we have people who**

16 **2→ stay ther:e, and then go home.**

17 (0.6)

18 CLI .hhh Some of the people there are (.) there for the same

19 reason and then they couldn't go ho:me anymore.=because

20 they need a lot of care. .hhh some of the people there

21 go to a [ nursing home.]

22 PAT [((coughs twice))]

23 CLI .HHH So I'm just letting you kno:w that our unit↑ is not

24 a one way unit.

25 (1.0)

26 CLI People are afraid when I ask them, (.) do you want to

27 come into hospital.

28 (.)

29 CLI So no::, I am‐ I‐ I give you that option because I can

30 see benefit for you going in there,

The clinician introduces the inpatient unit by referring generally to ‘people’ who receive care (line 6). As in the previous fragment, the use of general references makes their claim possible but not necessarily relevant to *this* patient.^48^, ^49^ In this instance, the clinician contradicts a possible understanding of inpatient palliative care as only being provided immediately before death by explaining the use of this unit for people at ‘various stages of their … palliative care journeys’ (arrow 1, lines 6 and 7). They also explain to the patient why active treatment in an inpatient setting is necessary (lines 9 and 10).

After explaining why a patient would be admitted for symptom management (lines 10 and 12–14), the clinician makes clear that people can be discharged following an admission (arrow 2, lines 15–16). The clinician explains that some may go to a nursing home if additional care is required to what is available at home (lines 18–21). These explanations are summarised by saying the unit ‘is not a one‐way unit’ (lines 23 and 24), which contradicts a possible understanding that people admitted to an inpatient palliative care unit will necessarily remain there until their death.

In addition to the focal practices, the clinician in this fragment also uses an explanation about fear to account for why she is managing possible understandings of inpatient palliative care being synonymous with care provided immediately before death (lines 26 and 27). General references are again used, which makes the clinician's explanation possible but not necessarily applicable to the patient. The clinician's categorisation of people as fearful follows an explanation that contradicts a possible understanding of inpatient palliative care as synonymous with care provided immediately before death. The positioning of these matters, one following the next, in the explanation facilitates a possible understanding of them as tied together.^50^, ^51^ In this case, the positioning infers that a ‘person's’ fear could be based—although again, not necessarily—on narrow understandings about palliative care.

## 4 DISCUSSION

### 4.1 Main findings

A core focus for palliative care is improving the quality of life of patients with life‐threatening illness,^2^ and can include active treatment.^2^, ^14^, ^15^ Nevertheless, evidence suggests that public understandings continue to narrowly focus on palliative care as being provided immediately before death.^4^, ^5^, ^6^, ^7^ The findings of the current study are consistent with existing research, which considered public understandings of palliative care through self‐report data such as surveys and interviews.^18^ By observing initial palliative care consultations, the current study finds narrow understandings of palliative care among the general public also appear relevant among patients who are referred to palliative care. The study findings indicate that because patients can ask questions about care that are consistent with a narrow understanding of palliative care, clinicians recurrently use practices that pre‐empt and contradict these narrow understandings.

### 4.2 What this study adds

The current study identifies two practices palliative care clinicians use to contradict possibly narrow understandings of palliative care. The first practice involved clinicians using extended turns‐at‐talk to explain inpatient palliative care as being used for a range of purposes, including active treatment, which challenges possible understandings that inpatient palliative care is only provided immediately before death. The second practice involved clinicians referring to the possibility of being discharged from the inpatient unit, which contradicts possible understandings that people admitted to the unit remain there until their death. These two practices seem to take into account a common understanding of palliative care as only being provided close to death.^4^, ^5^, ^6^, ^7^

General references were recurrently used when providing information that contradicts possible understandings of palliative care as synonymous with care provided immediately before death. Existing research suggests the majority of the general public associate palliative care with care provided immediately before death.^4^, ^5^, ^6^, ^7^, ^18^ However likely it may be, there is no guarantee that an individual patient will necessarily have this narrow understanding of palliative care. Making characterisations about people generally softens the direct relevance of the matter to the recipient.^48^, ^49^ For clinicians seeking to challenge, or correct, narrow understandings of palliative care, generalised claims provide a way of pre‐empting such understandings without asserting that an individual patient necessarily subscribes to them.

### 4.3 Limitations and future research

To identify exemplar communication practices of initial palliative care consultations, this study focused on specialist palliative care clinicians. Excluding clinicians who functionally deliver palliative care, such as some general practitioners, hospital ward staff or community nurses, means differences in communication practices when initially consulting about palliative care could not be assessed. Further recordings and analysis of general practitioners providing palliative care could deliver a deeper understanding of how patients' understandings of palliative care are initially managed.

## 5 CONCLUSION

When a patient is diagnosed with a life‐threatening illness, conversations about the future can be difficult.^19^, ^20^ A clinicians' confidence in managing these conversations could be a barrier to early referral to palliative care.^21^, ^22^, ^23^ Research suggests that referring clinicians are sometimes poorly equipped to manage patients' understandings of palliative care as more than care provided immediately before death.^8^, ^9^, ^10^ By virtue of primary treating teams' existing relationships with patients, these clinicians are well positioned to help patients understand the range of reasons they are being referred to palliative care. These findings highlight scope for greater partnership with primary treating teams who are referring patients to palliative care to inform patients about palliative care. Educational materials targeting clinicians could be created to provide information about how they might help patients and family members understand the range of reasons for referral to palliative care. Overall, the findings of this study show that pre‐emptively contradicting narrow public understandings of palliative care continue to be imperative for early referral and utilisation of holistic palliative care.

## AUTHOR CONTRIBUTIONS

**Holly Sansone**: Conceptualisation (supporting); funding acquisition (supporting); data curation; formal analysis (lead); validation; writing—original draft (lead), writing—review and editing (equal). **Stuart Ekberg**: Conceptualisation (lead); funding acquisition (lead); project administration; supervision; writing—original draft (supporting); formal analysis (supporting); validation; writing—review and editing (equal). **Sarah Lord**: Conceptualisation (supporting); writing—review and editing (equal). **James Stevenson**: Conceptualisation (supporting); funding acquisition (supporting); investigation (lead); writing—review and editing (equal). **Katherine Martinez**: Conceptualisation (supporting); funding acquisition (supporting); investigation; writing—review and editing (equal). **Patsy Yates**: Conceptualisation (supporting); funding acquisition (supporting); writing—review and editing (equal).

## CONFLICT OF INTEREST STATEMENT

The authors declare no conflict of interest.

## ETHICS STATEMENT

The study observed the principles of the Australian National Health and Medical Research Council's National Statement on Ethical Conduct in Human Research. Ethics clearance was obtained from the Prince Charles Hospital Human Research Ethics Committee on 2 February 2020 (LNR/2020/QPCH/60900), with administrative approval from the Queensland University of Technology Human Research Ethics Committee on 14 February 2020 (Approval reference: 2000000107). Consultations were recorded only if every person involved independently provided informed, written consent to participate.

## Supporting information

Supporting information.

## Data Availability

The data that support the findings of this study are available on request from the corresponding author. The data are not publicly available due to privacy or ethical restrictions. Ethical clearance for this study does not permit data sharing.
