# Supplementary material for: Managing understandings of palliative care as more than care immediately before death: Evidence from observational analysis of consultations
Source: Health Expect. 2023 Nov 5;27(1):e13903. doi: 10.1111/hex.13903 (PMC10726268; doi:10.1111/hex.13903)
Supplement: Supplementary file 1 — Supporting information. [file HEX-27-e13903-s001.docx]

# Appendix A

Transcription Conventions

*Temporal dimensions*

Wo[ rd ] Square brackets mark speaker overlap, with left square brackets

[Wo]rd indicating overlap onset and right square brackets indicating overlap offset.

Word=word An equals sign indicates absence of discernable silence between two utterances or actions, which can occur within a single person’s turn or between the turns of two people.

Word (0.4) word A number within parentheses refers to silence, which is measured to the nearest tenth of a second and can occur either as a pause within a current speaker’s turn or a gap between two speaker’s turns.

Word (.) word A period within parentheses indicates a micropause of less than two- tenths of a second.

*Verbal conduct*

Word. A period indicates falling intonation at the end of a unit of talk.

Word, A comma indicates slightly rising intonation.

Word¿ An inverted question mark indicates moderately rising intonation.

Word? A question mark indicates rising intonation.

Word Underlining indicates emphasis being placed on the underlined sounds.

Wo:::rd Colons indicates the stretching of the immediately preceding sound, with multiple colons representing prolonged stretching.

Wo::rd Underlining followed by one or more colons indicates a shift in pitch during the pronunciation of a sound, with rising pitch on the underlined component followed by falling pitched on the colon component that is not underlined.

Wo::rd An underlined colon indicates the converse of the above, with rising pitch on the underlined colon component.

↑Word↑ Upward arrows mark a sharp increased pitch shift, which begins in the syllable following the arrow. An utterance encased with upward arrows indicates that the talk is produced at a higher pitch than surrounding talk.

↓Word↓ Downward arrows mark a sharp decreased pitch shift, which begins in the syllable following the arrow. An utterance encased with downward arrows indicates that the talk is produced at a lower pitch than surrounding talk.

WORD Upper case indicates talk produced at a louder volume than surrounding utterances by the same speaker.

°Word° Words encased in degree signs indicate utterances produced at a lower volume than surrounding talk.

>Word< Words encased with greater-than followed by less than symbols indicate talk produced at a faster pace than surrounding talk.

Wor- A hyphen indicates an abrupt termination in the pronunciation of the preceding sound.

.hhh A period followed by the letter ‘h’ indicates audible inhalation, with more letters indicating longer inhalation.

(word) Words encased within single parentheses indicate an utterance that was unclear to the transcriptionist. Unfilled space within the parentheses indicates that it was not possible to identify a possible hearing of an utterance.
